# Ketogenic diet ameliorates attention deficit hyperactivity disorder in rats via regulating gut microbiota

**DOI:** 10.1371/journal.pone.0289133

**Published:** 2023-08-16

**Authors:** Yu Liu, Changhong Yang, Yingxue Meng, Yonghui Dang, Lin Yang

**Affiliations:** 1 Department of Pediatrics, The Second Affiliated Hospital of Xi’an Jiaotong University, Xi’an, Shaanxi, China; 2 College of Medicine and Forensics, Xi’an Jiaotong University Health Science Center, Xi’an, Shaanxi, China; Faculty of Agriculture, Al-Azhar University, EGYPT

## Abstract

Attention deficit hyperactivity disorder (ADHD) is a common mental behavioral disorder in children. Alterations in gut microbiota composition are associated with neurological disorders. We aimed to investigate whether a ketogenic diet (KD) can be an alternative therapy for ADHD by altering the gut microbiota. Male spontaneously hypertensive rats (SHR) and Wistar Kyoto (WKY) rats were randomly allocated to the normal diet (ND), methylphenidate (MPH), and KD groups. SHR in groups KD and MPH exhibited a significant increase in behavioral characteristics of ADHD, such as distance moved and immobility time. KD and MPH treatment led to a significant elevation in concentrations of 5-HT, AC, cAMP, and NE of brain tissue and the expression of DRD1, DAT, PKA, DARPP32, and cAMP at the protein level in WKY rats and SHR. KD and MPH significantly increased the richness and diversity of gut microbiota in SHR. The abundance of *Ruminococcus_gauvreauii_group*, *Bacteroides*, *Bifidobacterium*, and *Blautia* significantly increased, whereas that of *Lactobacillus*, *Romboutsia*, *Facklamia*, and *Turicibacter* significantly declined in the KD group compared with the ND group. The gut microbiota in the KD group of SHR mainly participated in amino acid metabolism- and sugar metabolism-related pathways. KD might alleviate behavioral disorders in ADHD by regulating gut microbiota. This study provides novel insights for the use of KD in treating ADHD.

## Introduction

Attention deficit hyperactivity disorder (ADHD) is a mental disorder of childhood that manifests as persistent inattention, hyperactivity, and impairment of social and academic functions [1]. The pathogenesis of ADHD may be related to neurotransmitter-related disorders, such as abnormal expression of dopamine (DA), 5-hydroxytryptamine (5-HT), and norepinephrine (NE) [2–4]. Methylphenidate (MPH) is the first-line drug in the treatment of ADHD, but it cannot improve all symptoms. Approximately 30% of the children did not respond adequately to the drug or could not tolerate its side effects, such as loss of appetite and twitch, and the symptoms reappeared after drug withdrawal [5]. Therefore, it is of great significance to find ideal drugs for ADHD treatment.

Gut microbiota is important for health and immunity and is linked to neurological disorders such as Alzheimer’s disease, autism, Parkinson’s disease, and ADHD [6, 7]. The composition and proportion of intestinal microbiota in patients with ADHD are unbalanced. Jiang et al. analyze the composition of gut microbiota in 51 adolescent patients with ADHD and 32 healthy controls from China and report that *Faecalibacterium* is significantly reduced in patients with ADHD [8]. Evidence has find that alpha diversity of gut microbiota in adolescent patients with ADHD is lower than that in controls and is negatively correlated with ADHD symptom scores [9]. Recently, it has been gradually recognized that gut microbiota can regulate the central nervous system through the microbiota–gut–brain axis and affect the occurrence and development of behavioral disorders [10]. Evidence demonstrates that gut microbiota plays a principal part in neurotransmitter transmission and neuroplasticity [11]. However, there is less study on the microbiota–gut–brain axis in the development of ADHD. Understanding the interaction between the microbiota–gut–brain axis and ADHD may open new avenues for early intervention and treatment.

The ketogenic diet (KD) contains high fat, low carbohydrate, and moderate protein [12]. KD can change the dietary structure, such that cells no longer use glucose for energy, forcing the body to use ketone bodies as an alternative energy source, thereby protecting the function of various tissues and organs [13]. KD plays a central role in neurological diseases such as epilepsy, Alzheimer’s disease, migraine, and Parkinson’s disease [14]. KD improve ADHD-like behavior in dogs with idiopathic epilepsy [15]. Recent studies have found that gut microbiota mediate the therapeutic effects of KD [16]. KD ameliorate colitis by decreasing colonic group 3 innate lymphoid cells through the regulation of gut microbiota [17]. In an autism spectrum disorder mouse model, KD alleviate neurological symptoms potentially through alternation in the gut microbiome [18]. However, studies showing that KD improves ADHD through the microbiota–gut–brain axis are lacking.

The behavioral characteristics of spontaneously hypertensive rats (SHR) are similar to those of ADHD, and SHR is the most widely studied animal model of ADHD [19]. In this study, we compared oral KD with MPH treatment to evaluate whether KD could be an alternative therapy for ADHD. We aimed to determine whether KD improved ADHD symptoms and investigate the neurotransmitter expression and gut microbiota profile in SHR and WKY rats to understand the role of KD in improving ADHD.

## Experimental procedure

### Experimental animals

Four-week-old male SHR and Wistar Kyoto (WKY) rats were purchased from Nanjing Junke Bioengineering Company. Rats were kept in specific pathogen-free rooms at 20°C–22°C, 12-h light/12-h dark, and relative humidity of 60%–70% and fed *ad libitum*.

**Fig 1:**
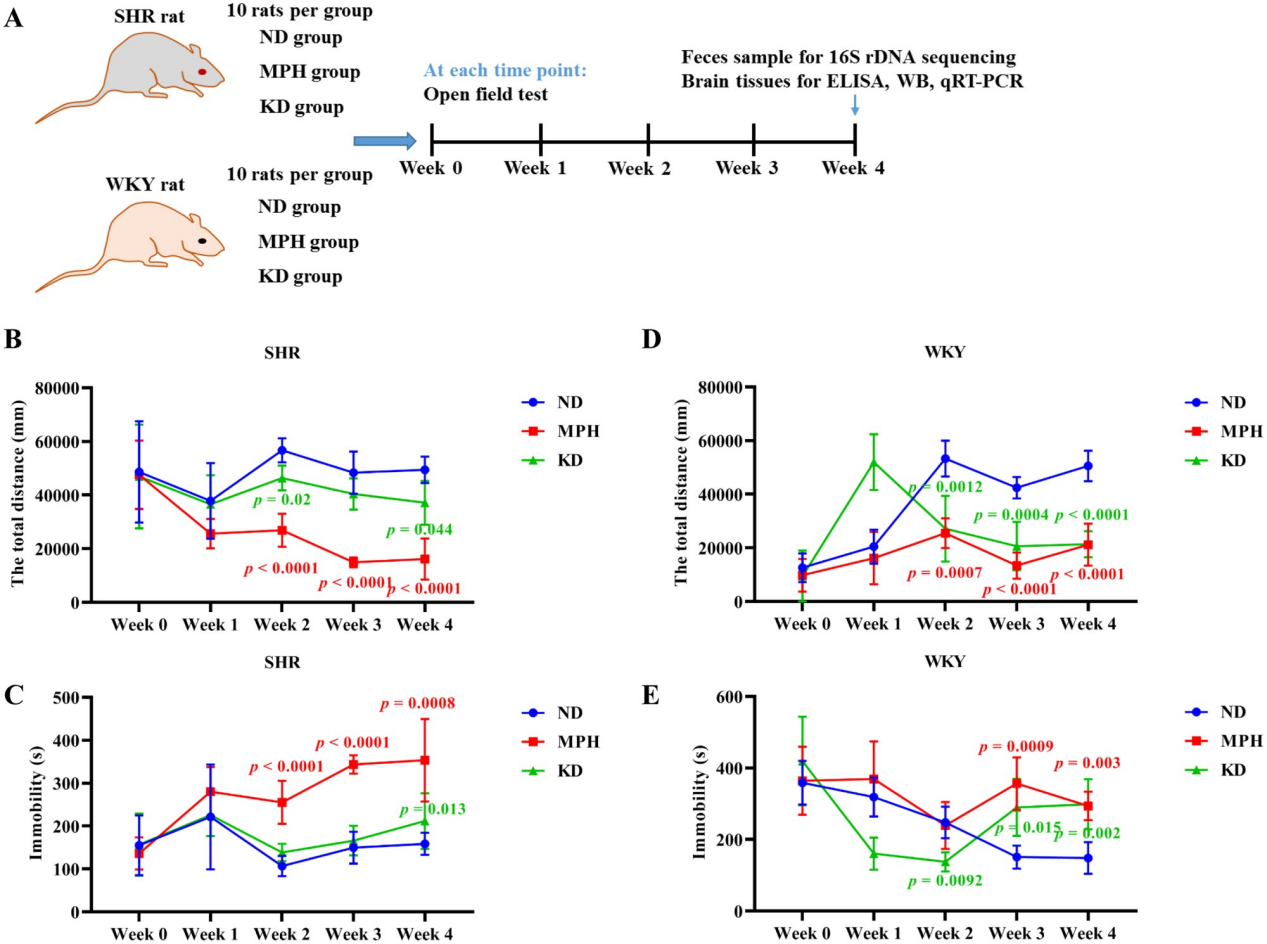
Ketogenic diet improves ADHD in SHR. (A) Time flow diagram of the experimental design. (B) Total distance moved by spontaneously hypertensive rats (SHR) in the open field test. (C) Immobility time of SHR in the open field test. (D) Total distance moved by Wistar Kyoto (WKY) rats in the open field test. (E) Immobility time of WKY rats in the open field test. ND represents normal diet. MPH represents methylphenidate. KD represents ketogenic diet. Data are presented as mean ± SD. *p* values in red font indicate MPH *vs*. ND; *p* values in green font indicate KD *vs*. ND.

The experimental design is illustrated in Fig 1A. SHR were used as experimental groups and WKY rats served as normal control groups. Both SHR and WKY rats were randomly assigned to three groups with 10 rats in each group: KD, MPH, and normal diet (ND) groups. Rats in the KD group was fed KD for 28 d. Rats in the MPH group was fed with ND, and were given MPH at the dose of 1.5 mg/kg daily by gavage for 28 d. Rats in the ND group was fed with ND for 28 d.

### Sample collection

On weeks 0, 1, 2, 3, and 4, rats were subjected to the open field test. At week 4 (day 28), feces and brain tissues were collected. The rats were euthanized by injecting 1% pentobarbital (50 mg/kg) intraperitoneally. Feces and brain tissues were stored at −80°C until use. This study was approved by the Ethics Committee of The Second Affiliated Hospital of Xi’an Jiaotong University.

### Open field test

Open field tests are used to analyze movement, anxiety, and grooming in rodents [20]. Changes in motor behavior can indicate changes in neural processes that reflect abnormal brain function [21]. A multi-unit opening box of 100 cm × 100 cm × 40 cm was used: each independent opening box measured 50 cm × 50 cm × 40 cm. The camera was located 160 cm above the center of the experimental box to monitor four independent opening boxes. The rats were gently placed in the central area of the experimental box, and the distance moved, speed of movement, and distance moved from the center were recorded with a software. The camera tracked rat movement. The open field test was performed on days 0, 7, 14, 21, and 28 after rats were fed with the test diet. Each experiment lasted for 5 min.

### Enzyme-linked immunosorbent assay (ELISA)

The levels of 5-HT (ml028308, China), adenylate cyclase (AC; ml714032, China), cyclic adenosine monophosphate (cAMP; ml002907, China), and NE (ml791400, China) in brain tissue were measured using ELISA kits (Shanghai Enzyme-Linked Biotechnology Co. Ltd., China) in accordance with the manufacturer’s instructions. The absorbance value of each well was tested at 450 nm using a Microplate Reader (Thermo Scientific, USA).

### Real-time quantitative PCR (qRT-PCR)

Total RNA was isolated from brain tissues using TRIzol reagent (Invitrogen, USA). After the detection of RNA quality and purity, the Reverse Transcription kit (Thermo, USA) was employed to reverse-transcribe RNA into cDNA. qRT-PCR was conducted using the SYBR Green PCR kit (Roche, Switzerland) on Applied Biosystems Q6 real-time PCR system (Applied Biosystems, CA). The reaction conditions were as follows: 95°C for 10 min followed by 40 cycles of 95°C for 15 s and 60°C for 60 s. GAPDH was taken as the internal reference. The 2^−ΔΔCt^ method was utilized to detect the relative mRNA expression of dopamine receptor D1 (DRD1), dopamine transporter (DAT), PKA, DARPP32, and cAMP. The primer sequences are shown in S1 Table.

### Western blot

Total protein was extracted from the brain tissues of WKY rats and SHR using RIPA lysis buffer (Thermo, USA). The concentration of total protein was measured with a BCA kit (Thermo Scientific, USA). Approximately 20 μg of protein was isolated through 10% SDS-PAGE and transferred to polyvinylidene fluoride membranes. The membranes were stained with Ponceau (Acros Organics) to confirm successful protein transfer. Whereafter, the membranes were blocked with TBST solution containing 5% milk at room temperature for 3 h, and then incubated with primary antibodies against DRD1 (1:1000; ab279713, Abcam, UK), DAT (1:1000; ab184451, Abcam, UK), PKA (1:5000; ab32390, Abcam, UK), DARPP32 (1:1000; ab40801, Abcam, UK), cAMP (1:20000; ab76238, Abcam, UK), and GAPDH (1:2000; 60004-1-Lg, Proteintech, USA) overnight at 4°C. Subsequently, the membranes were washed with 1% TBST solution, incubated with goat anti-mouse IgG H&L (HRP) (1:1000; ab205719, Abcam, UK) at room temperature for 1.5 h. In the end, after washing three times with 1% TBST solution, blots were exposed to an ECL luminescence kit (Thermo, USA). Image J (v5.2.1) was employed to analyze the bands.

### 16S rDNA sequencing and bioinformatics analysis

Total DNA from feces was isolated using QIAamp 96 PowerFecal QIAcube HT Kit (Qiagen). DNA quality was examined by 1% agarose gel electrophoresis and DNA concentration was measured with a NanoDrop 2000 (NanoDrop Technologies, USA). The V3–V4 region of the 16S rDNA gene was PCR amplified using primers 338F (5′-ACTCCTACGGGAGGCAGCAG-3′) and 798R (5′-GGACTACHVGGGTWTCTAAT-3′). The PCR product was extracted with 2% agarose gel electrophoresis and purified with AxyPrep DNA Gel Extraction Kit (Axygen Biosciences, USA) in accordance with the manufacturer’s instructions. The PCR product was quantified with QuantiFluor-ST (Promega, USA). Finally, the Illumina MiSeq PE300 platform was used for 16S rDNA sequencing.

The paired-end raw reads were filtered with Trimmomatic (version 0.35). The filtered reads were merged using Flash (version 1.2.11). To obtain clean tags, raw tags were percolated by QIIME (version 1.9.1). The sequences were allocated to operational taxonomic units (OTUs) using UCLUST (Version 1.2.22). OTUs clustering were conducted with a 97% similarity threshold using UPARSE software (version 7.0.1090). The Silva database (Release138) was used to divide OTUs into taxa. Alpha diversity (ACE, Simpson, and Shannon indexes), and principal component analysis (PCA) were carried out with QIIME (version 1.9.1). Venn diagram was utilized to exhibit the number of microbiota in different groups using R (version 1.6.16). Linear discriminant analysis (LDA) effect size (LEfSe) was used for differential abundance analysis of bacteria between groups. Functional prediction of bacterial communities was performed using PICRUSt.

### Statistical analysis

Statistical analyses were conducted with GraphPad Prism 9.0, and the data was exhibited as mean ± standard deviation (SD). Comparisons between more than two groups were performed by one-way ANOVA followed by Tukey’s *post hoc* test. *p* < 0.05 was deemed to be statistically significant.

## Results

### Ketogenic diet improves the behavioral symptoms of ADHD

To evaluate the effect of KD on behaviors of ADHD, the open field test was used to determine the total distance moved and immobility time in SHR. Expectedly, as a first-line drug in the treatment of ADHD, MPH significantly improved behavioral indicators of ADHD in SHR, as evidenced by shorter total distance and longer immobility duration compared with the ND group (Fig 1B and 1C). Similar efficacy to MPH was observed in the KD treatment group. The results showed that after 4 weeks of KD treatment, SHR in the KD group had a significantly shorter total distance (Fig 1B), and a significantly longer immobility duration (Fig 1C) than that in the ND group. Notably, in the KD group, total distance showed a significant downward trend at week 2 post-treatment, suggesting that KD may show behavioral effects after 2 weeks of treatment (Fig 1B). MPH and KD treatment significantly shortened total distance moved (Fig 1D) and increased immobility time (Fig 1E) in WKY rats, suggesting that MPH and KD have some sedative effect on WKY rats. Taken together, these results indicate that KD could relieve the behavioral characteristics of ADHD with hyperactivity, as well as impulsivity in SHR.

### Ketogenic diet increases neurotransmitter expression

**Fig 2:**
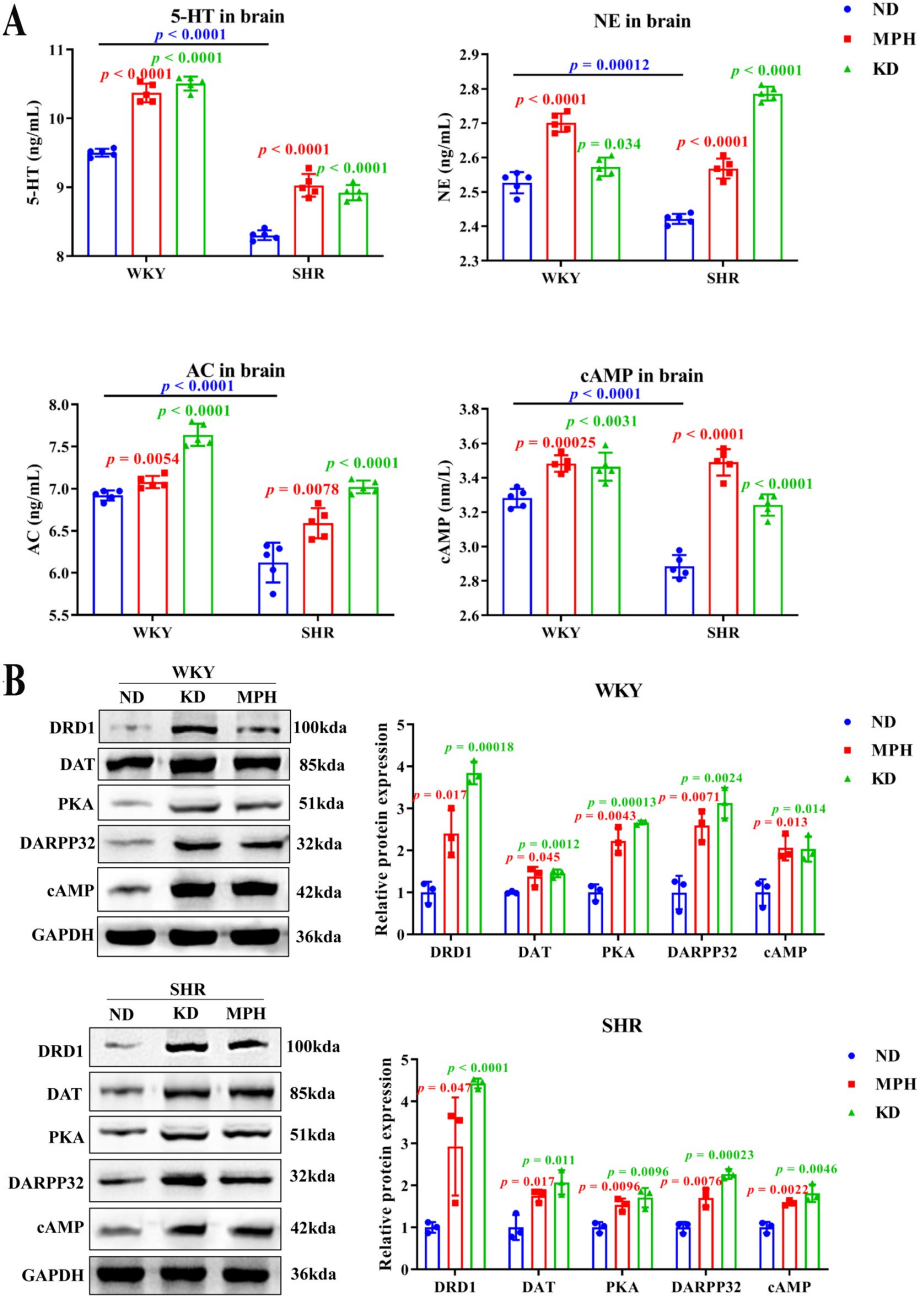
Ketogenic diet regulates the expression of neurotransmitters and DA-related genes in brain tissues. (A) The levels of 5-HT, NE, cAMP, and AC in the brain tissues of WKY rats and SHR were measured by ELISA. (B) Protein expression of DRD1, DAT, PKA, DARPP32, and cAMP in the brain tissues of WKY rats and SHR were tested with western blot. Data are exhibited as mean ± SD. *p* values in red font indicate MPH *vs*. ND; *p* values in green font indicate KD *vs*. ND; *p* values in blue font indicate WKY rats compared with SHR in the ND group.

To investigate the effect of KD on brain nervous system, we determined the effect of KD on the expression of neurotransmitters, including biogenic amines 5-HT and NE, and their downstream G protein effectors and second messengers, such as AC and cAMP, respectively [22]. ELISA analysis of brain tissues showed that the concentrations of 5-HT, NE, AC, and cAMP were significantly lower in SHR than WKY rats (Fig 2A), which is consistent with other studies [23, 24]. Moreover, compared with the ND group, a significant boost in the concentrations of 5-HT, NE, AC, and cAMP in the MPH and KD group were observed, both in SHR and WKY rats (Fig 2A). These results implicate that KD exhibits MPH-like efficacy and can increase the expression of neurotransmitters.

In addition, we also measured the expression of DA-related genes using WB and qRT-PCR, such as DA transporter (DAT), DA receptors (DRD1 and DARPP32), as well as downstream targets of DARPP32, including cAMP and PKA. WB analysis revealed that compared with the ND group, KD and MPH significantly upregulated the protein expression of DA-related genes (DRD1, DAT, PKA, DARPP32, and cAMP) in WKY rats and SHR (Fig 2B). However, KD had a statistically significant effect on the mRNA levels of only DRD1 in SHR (S1 Fig), suggesting that the regulation of DA-related genes by KD occurs at the protein level.

### Ketogenic diet alters the diversity of gut microbiota

**Fig 3:**
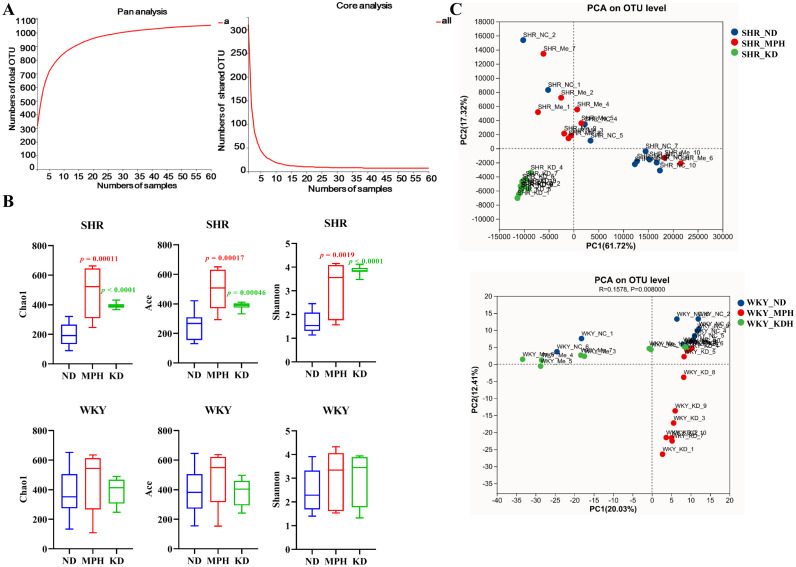
Ketogenic diet changes the diversity of gut microbiota in SHR. (A) Pan analysis curve and core analysis curve. (B) Chao1, ACE, and Shannon indexes in ND, MPH, and KD groups of WKY rats and SHR. (C) Principal component analysis (PCA) in groups ND, MPH, and KD of WKY rats and SHR. Data were shownare presented as mean ± SD. *p* values in red font indicate MPH *vs*. ND; *p* values in green font indicate KD *vs*. ND.

Studies have shown that the imbalance of gut microbiota is closely associated with ADHD [7]. Therefore, 16S rDNA sequencing was performed to evaluate the role of KD in the gut microbiota in SHR. Species abundance curves obtained from Pan/Core analysis based on annotation of the species classification of OTUs can be used to assess the adequacy of sequencing sample size. As shown in Fig 3A, Pan/Core analysis elaborates a flattening curve, indicating that the sample size was sufficient for sequencing. In total, 1005 and 933 OTUs were acquired in WKY rats and SHR, respectively (S2 Fig). Alpha diversity was evaluated by the Chao1, Ace, and Shannon indexes. In SHR, the Chao1, Ace, and Shannon values were significantly elevated after KD and MPH administration, compared with the ND group (Fig 3B), suggesting that KD and MPH increased community richness and diversity. However, no difference in the three indexes was observed between group ND and groups KD and MPH in WKY rats. PCA was employed to determine beta diversity of gut microbiota. Unweighted PCA analysis based on the OUTs level indicated a clear separation between groups ND, KD, and MPH both in SHR and WKY rats (Fig 3C). Importantly, microbial communities were obviously separated between the ND and KD groups in SHR, whereas they were not separated between MPH and ND groups, indicating that the structure of the gut microbiota changed more obviously after KD treatment. Taken together, KD treatment altered gut microbiota diversity in SHR.

### Ketogenic diet improves intestinal microbiome imbalance

**Fig 4:**
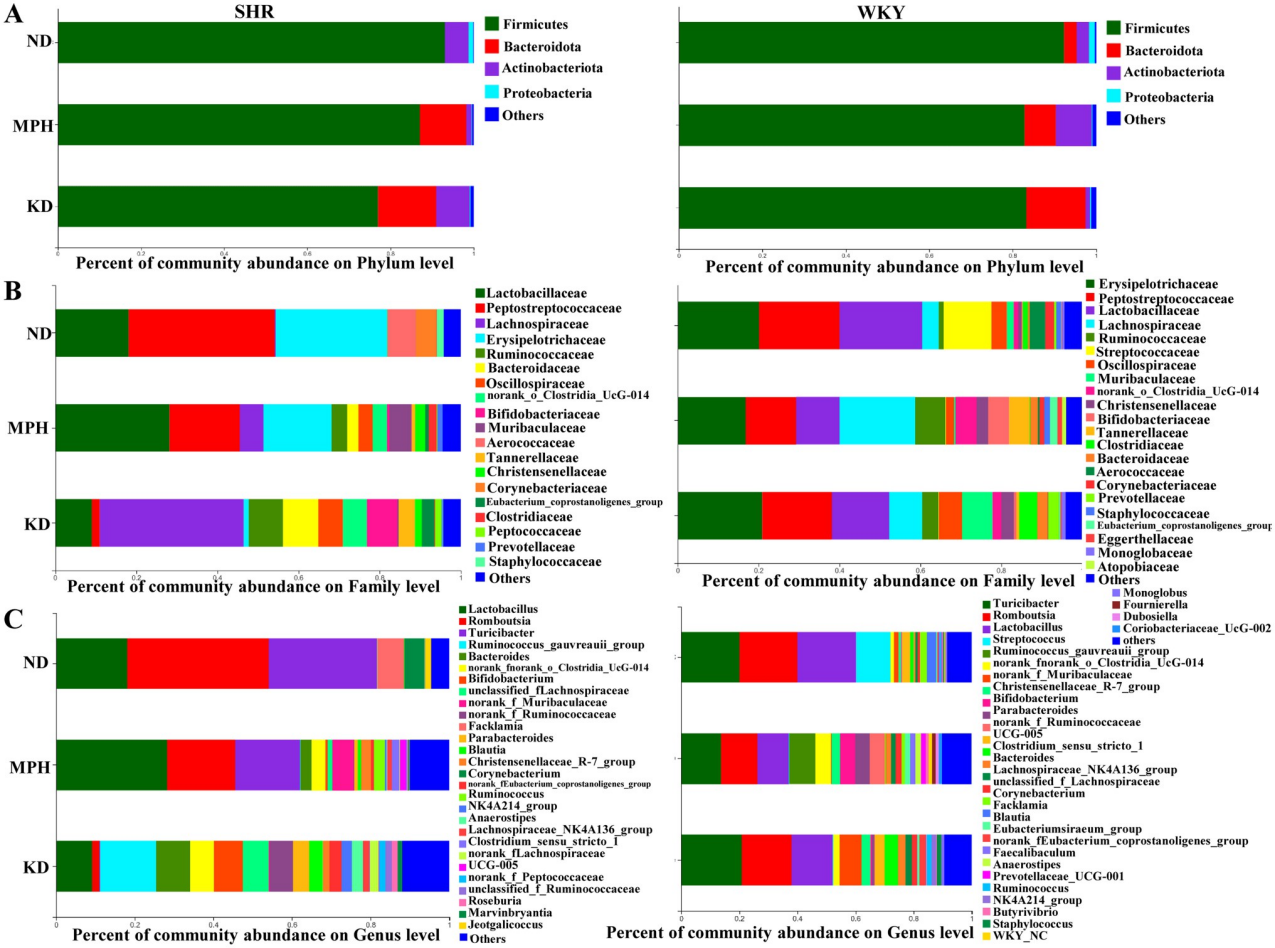
Ketogenic diet changes the structures of gut microbiota in SHR. (A) Gut microbiota composition in WKY rats and SHR at the phylum level. (B) Gut microbiota composition in WKY rats and SHR at the family level. (C) Gut microbiota composition in WKY rats and SHR at the genus level.

To examine the effect of KD on the structure of gut microbiota in SHR, the composition of gut microbiota in each group was investigated at the phylum, family, and genus levels. At the phylum level (Fig 4A), in both SHR and WKY rats, Firmicutes was the dominant population in all three groups. The Bacteroidota population in SHR almost disappeared compared with WKY rats in the ND group. However, compared with the ND group, KD and MPH treatment resulted in a substantial change in the abundance of Bacteroidota from none to dozens, increasing by approximately 10% in SHR. These results indicate that KD treatment favored Bacteroidota survival and expansion to improve ADHD.

At the family level (Fig 4B), in the ND group, the abundance of Peptostreptococcaceae was increased and that of Lachnospiraceae was decreased and almost disappeared in SHR, whereas the Streptococcaceae population was completely lost, compared with WKY rats. In SHR, KD and MPH treatments significantly reversed this alteration, as indicated by a decrease in PEP abundance and an increase in LAC abundance, compared with the ND group. Interestingly, the loss of Streptococcaceae occurred not only in SHR but also in KD-treated healthy WKY rats, implying that KD has a greater killing effect on Streptococcaceae populations. Overall, these results suggest that KD treatment may play a role in improving ADHD at the family level mainly by affecting Peptostreptococcaceae and Lachnospiraceae.

At the genus level (Fig 4C), in the ND group, the abundance of *Romboutsia*, *Turicibacter*, *Corynebacterium*, and *Facklamia* increased and that of *Streptococcus*, *UCG-005*, and *Clostridium_sensu_stricto_1* decreased in SHR, compared with WKY rats. In SHR, the abundance of *Romboutsia*, *Turicibacter*, and *Facklamia* decreased in the KD group compared with the ND group. In stark contrast to the ND group, microbial diversity clearly enhanced after KD or MPH treatment, which is consistent with the results shown in Fig 4B. These results imply that KD treatment may lead to a loss of dominant populations at the genus level and support the survival of more rare populations.

### Analysis of differentially abundant gut microbiota

**Fig 5:**
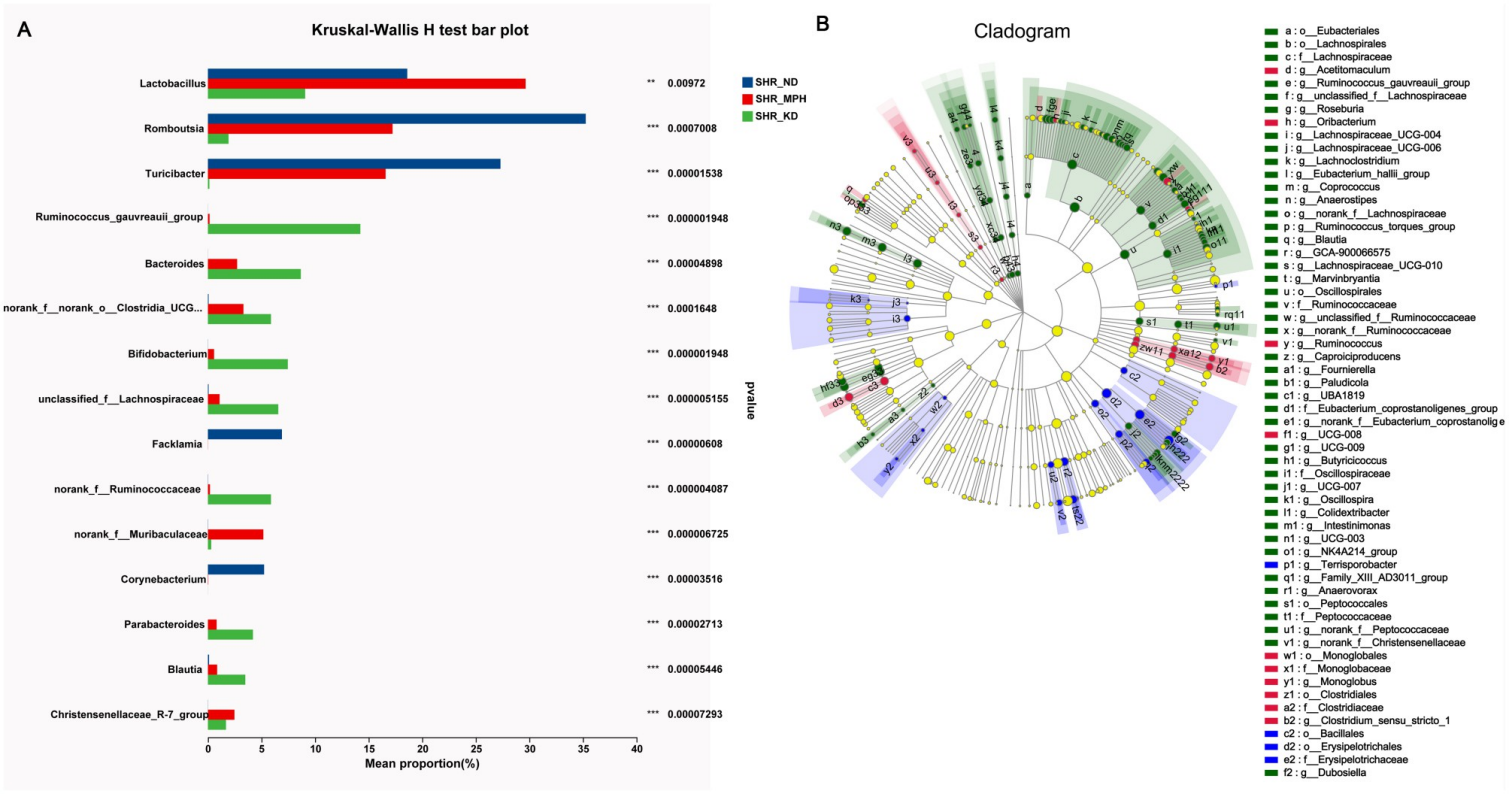
Differential abundance analysis of gut microbiota in SHR. (A) Differentially abundant microbiota between the three groups in SHR was tested by the Kruskal–Wallis *H* test. (B) Identification of the differentially abundant microbiota at multilevels by LEfSe analysis. o: order, f: family, g: genus.

To identify microbiota with differential abundance in response to KD treatment in SHR, we performed group difference analysis and multilevel species difference analysis. The Kruskal–Wallis *H* test was used to determine differences in the proportions of gut microbiota between ND, MPH, and KD groups. The results showed that the abundance of *Lactobacillus*, *Romboutsia*, *Turicibacter*, *Facklamia*, and *Corynebacterium* was significantly reduced, whereas that of *Ruminococcus_gauvreauii_group*, *Bacteroides*, *Bifidobacterium*, *Parabacteroides*, and *Blautia* was significantly increased in the KD group compared with the ND group (Fig 5A). Whereafter, LefSe analysis was utilized to determine differentially abundant microbiota in each group at multilevel. At the family level, Lachnospiraceae, Ruminococcaceae, Peptococcaceae, and Oscillospiraceae were enriched in the KD group, whereas Erysipelotrichaceae was enriched in the ND group (Fig 5B). At the genus level, *Terrisporobacter* was enriched in the ND group, and most of the rest, including *Roseburia*, *Lachnoclostridium*, *Coprococcus*, and *Anaerostipes*, were enriched in the KD group. Taken together, KD led to remarkable changes in the gut microbiota of SHR. The differential flora in response to KD treatment may be significant in improving the symptoms of ADHD.

### Prediction of biological function of gut microbiota

**Fig 6:**
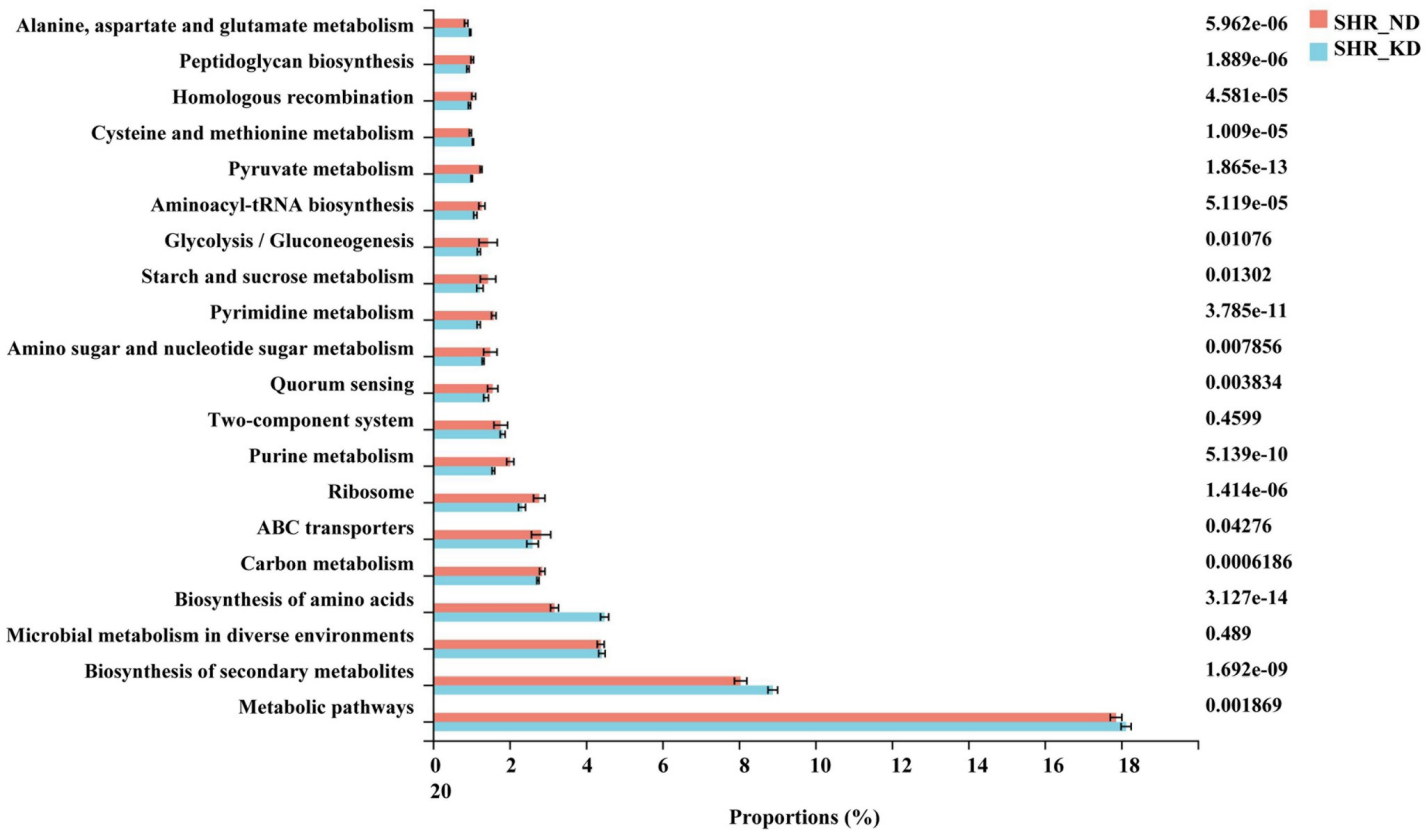
Comparison of the top 20 KEGG pathways between the ND and KD groups in SHR.

To characterize the functions of gut microbiota in SHR, we performed PICRUSt analysis. The top 20 microbial functions were estimated at level 1 of the KEGG pathway (Fig 6). The results showed that compared with the ND group, the KD group exhibited significantly increased abundance in “Alanine, aspartate and glutamate metabolism,” “Cysteine and methionine metabolism,” and “Biosynthesis of amino acids,” suggesting that KD administration improves ADHD by promoting gut microbiota-mediated amino acid metabolism. In addition, we also observed that the abundance of sugar metabolism-related pathways, such as “Peptidoglycan biosynthesis,” “Pyruvate metabolism,” “Glycolysis/Gluconeogenesis,” and “Starch and sucrose metabolism,” were significantly reduced in the KD group compared with the ND group. Thus, KD treatment impaired sugar metabolism in SHR.

## Discussion

Although MPH is the first-line drug for the treatment of ADHD, it has significant adverse effects and limited therapeutic efficacy. Therefore, there is an urgent need to investigate new therapeutic agents for ADHD. Our findings have shown that KD could relieve behavioral symptoms of ADHD in SHR, increase neurotransmitter expression, and increase protein expression of DA-related genes (DRD1, DAT, PKA, DARPP32, and cAMP). KD increased the richness and diversity of the gut microbiota in SHR at the phylum, family, and genus levels. Importantly, we identified a large number of microbiotas with significantly differential abundance after KD treatment at multiple levels. We found that gut microbiota between ND and KD groups in SHR were mainly involved in amino acid metabolism- and glucose metabolism-related pathways.

DA, 5-HT, and NE are important neurotransmitters of the nervous system related with the development of ADHD. A disorder in one or more links of the neurotransmitter network can lead to the disordering of the entire network, and ultimately, to the occurrence of ADHD. In our study, the concentrations of 5-HT, NE, AC, and cAMP of SHR in the KD group were strikingly higher than that in the ND group. These results are consistent with previous two studies [23, 24]. Moreover, we also proved that the expressions of DRD1, DAT, cAMP, PKA, and DARPP32 were upregulated after KD treatment. DRD1 is a DA receptor and DAT is a DA transporter: both exert important functions in the DA system. An animal study has shown that DRD1 regulates the prefrontal striatum pathway, and thus, plays a therapeutic role in ADHD [25]. Wang et al. report that baicalein significantly increase the content of AC, cAMP, and PKA in the prefrontal cortex and improve behavioral performance, such as hyperactivity and impulse, in SHR [26]. Napolitano et al. confirm that amphetamine have a motor calming effect by increasing the concentration of DA in the brain and activating the DRD1/cAMP/PKA/DARPP32 signaling pathway [27]. Therefore, KD may play a therapeutic role in ADHD through the neurotransmitter-mediated DRD1/cAMP/PKA/DARPP32 pathway.

Patients with ADHD have an imbalance in the composition and proportion of intestinal flora. Gut microbiota plays a central part in ADHD by affecting the synthesis and metabolism of neurotransmitters, such as DA, 5-HT, and NE, as well as their precursors [28]. Prehn-kristensen et al. found that the alpha diversity of the intestinal microflora was reduced in adolescent patients with ADHD, and the ADHD symptom score was negatively correlated with alpha diversity [9]. Consistently, Wang et al. also discover that the gut microbiota communities in patients with ADHD exhibited remarkably increased Shannon and Chao indexes relative to the controls [29]. In this study, KD enhanced the alpha diversity of the gut microbiota in SHR, indicating that KD can improve ADHD by regulating gut microbiota diversity.

Changes in intestinal flora can affect the development and function of the nervous system through the gut–brain axis, and then lead to the occurrence and development of ADHD [28]. In this study, the abundance of *Ruminococcus_gauvreauii_group*, *Bacteroides*, and *Bifidobacterium* increased in the KD group relative to the ND group in SHR. *Bifidobacterium* produces gamma-aminobutyric acid (GABA), which is the primary repressive neurotransmitter in the human cerebral cortex. GABA levels in children with ADHD is reportedly low and negatively correlated with impulsive behavior [30]. *Bifidobacterium* also participates in the DA neural reward system, suggesting that *Bifidobacterium* is a potential biomarker for ADHD [31, 32]. *Ruminococcus*, *Blautia*, and *Bacteroides* can produce short-chain fatty acids (SCFAs) [33]. SCFAs have neuroactive and anti-inflammatory effects on the host [34]. Supplementation with SCFAs can improve impaired microglia function in germ-free mice [35]. Evidence suggests that intestinal flora can influence the level of brain derived neurotrophic factor by producing SCFAs; germ-free mice with reduced brain derived neurotrophic factor levels developed working memory problems [36]. *Bacteroides* reportedly correlate with levels of hyperactivity and impulsivity in ADHD [37]. Therefore, KD maybe improve ADHD through the gut microbiota–gut–brain axis.

In our study, KD treatment increased the relative abundance of amino acid metabolism-related pathways compared with the ND group in SHR. Patients with ADHD reportedly have lower levels of amino acids, including tryptophan (precursor of serotonin) and tyrosine (precursor of catecholamines) [38, 39]. Studies have suggest that amino acid supplementation is mildly effective in ADHD [40, 41]. For example, DL-phenylalanine supplementation have a significant improvement in mood and mood lability in adult hyperactivity [42], and *S*-adenosyl-L-methionine supplementation significantly improves ADHD [43]. Although all three studies had a short-term benefit in amino acid supplementation, they illustrate the importance of amino acid metabolism in ADHD. Therefore, we suspected that KD administration would improve ADHD by promoting gut microbiota-mediated amino acid metabolism. Moreover, we found that sugar metabolism-related pathways were lower in the KD group compared with the ND group in SHR. Several studies have demonstrate an association between intake of dietary sugar or refined carbohydrate and clinically undesirable behaviors in patients with ADHD [44, 45]. Unfortunately, sugar restriction alone does not improve ADHD [46]. Beela and Raji prove that replacing bakery confectionaries, chocolates, soft drinks, maida, and junk food in the diet with the Recommended Daily Allowance significantly reduced ADHD symptoms in children [47]. Therefore, we believe that KD administration may improve ADHD by restricting sugar uptake. Taken together, amino acid metabolism and sugar intake restriction may be potential mechanisms for KD to improve ADHD.

## Conclusions

In conclusion, KD improved typical behavioral performance of ADHD with hyperactivity and impulsivity in SHR. Additionally, KD significantly activated the DRD1/cAMP/PKA/DARPP32 pathway in SHR. The richness and diversity of gut microbiota were altered in SHR after KD treatment, with significant changes in *Ruminococcus_gauvreauii_group*, *Bacteroides*, *Bifidobacterium*, and *Blautia* at the genus level. KD-induced gut microbiota were enriched in amino acid metabolism- and sugar-related pathways. These results give novel insights into the mechanism of KD in ADHD treatment.

## Supporting information

S1 Fig (TIF)

S2 Fig (TIF)

S1 Table Primers information used in this study. (DOCX)

S1 Raw image (PDF)
