# Supplementary figures and images for: Ketogenic diet ameliorates attention deficit hyperactivity disorder in rats via regulating gut microbiota
Source: PLoS One. 2023 Aug 16;18(8):e0289133. doi: 10.1371/journal.pone.0289133 (PMC10431618; doi:10.1371/journal.pone.0289133)

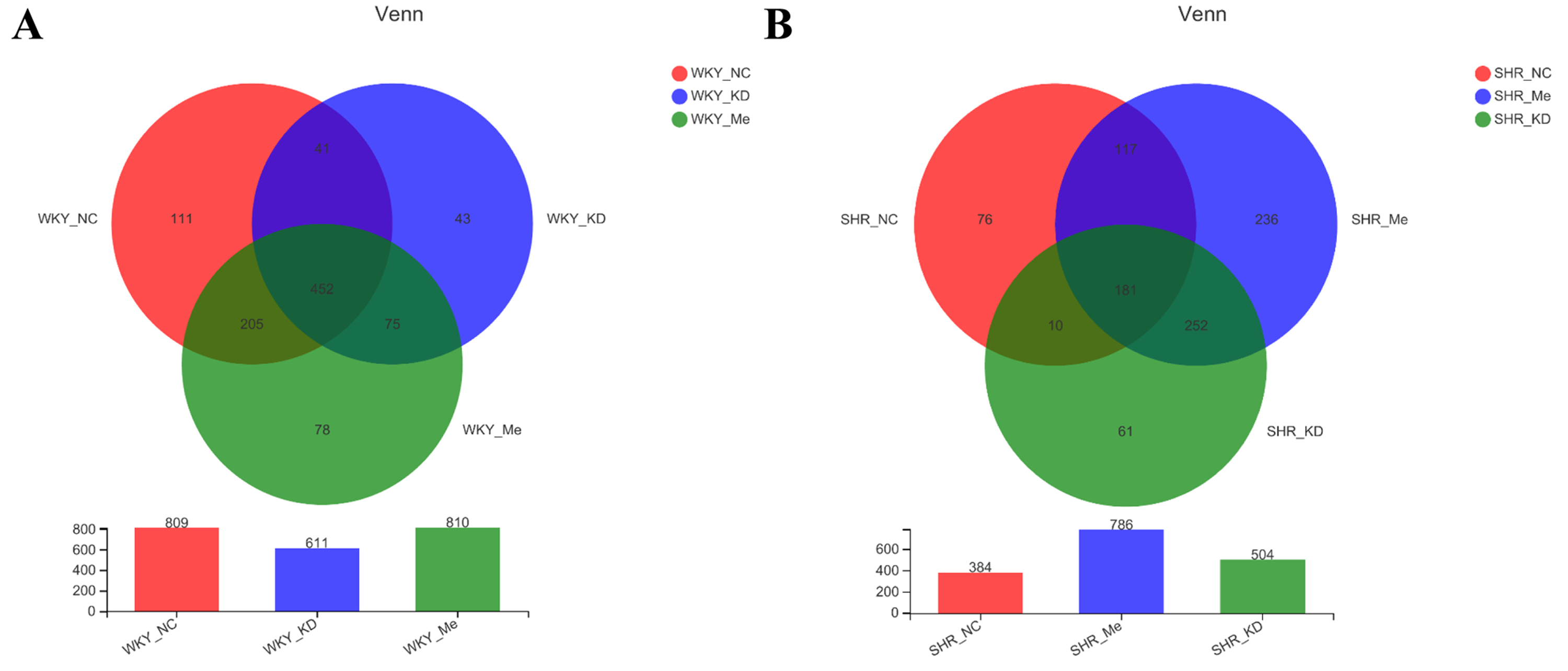

Supplement: S2 Fig — (TIF) [file pone.0289133.s002.tif]
