# Supplementary material for: Ketogenic diet ameliorates attention deficit hyperactivity disorder in rats via regulating gut microbiota
Source: PLoS One. 2023 Aug 16;18(8):e0289133. doi: 10.1371/journal.pone.0289133 (PMC10431618; doi:10.1371/journal.pone.0289133)
Supplement: S1 Table — (DOCX) [file pone.0289133.s003.docx]

| **Gene** | **Sequence of primer (5'to3')** |
| --- | --- |
| R-GAPDH-F | TGGAGAAACCTGCCAAGTATGAT |
| R-GAPDH-R | TCAAAGGTGGAAGAATGGGAGT |
| rno-DRD1-F | TCGAACTGTATGGTGCCCTT |
| rno-DRD1-R | AAGAATTCGCCCACCCAAAC |
| rno-DAT-F | GGACCTGGGCTCATCTTCAT |
| rno-DAT-R | GTCGATACCCAGAGTGAGCA |
| rno-PKA-F | TGGACGCCATGTTTGAAAAGA |
| rno-PKA-R | GTTCTCCAAAACTGCCACGG |
| rno-DARPP32-F | TTCCGGGTCTCAGAGCATTC |
| rno-DARPP32-R | CACAGGGGTTGGGTCTCTTC |
| rno-cAMP-F | TGAGGACCCAGATACTCCCA |
| rno-cAMP-R | GACTGTCCCCATACACTGCT |

**S1 Table. Primers information used in this study.**
