# Supplementary material for: Ketogenic diet ameliorates attention deficit hyperactivity disorder in rats via regulating gut microbiota
Source: PLoS One. 2023 Aug 16;18(8):e0289133. doi: 10.1371/journal.pone.0289133 (PMC10431618; doi:10.1371/journal.pone.0289133)

SHR cAMP

WKY cAMP

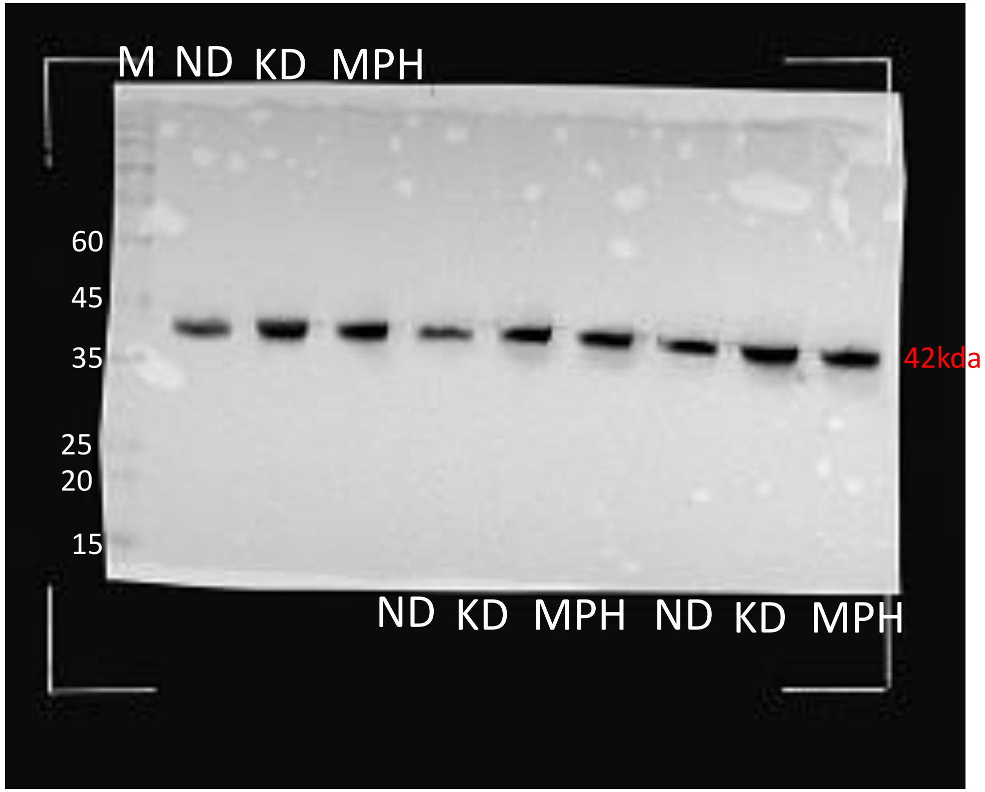

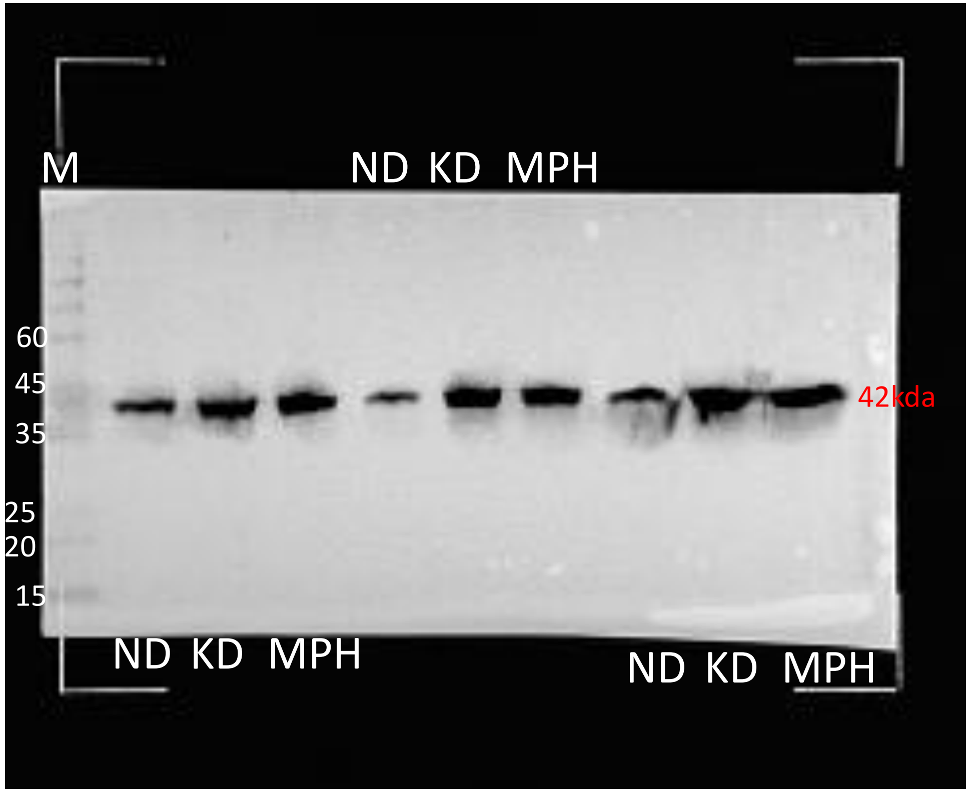

SHR DARPP32

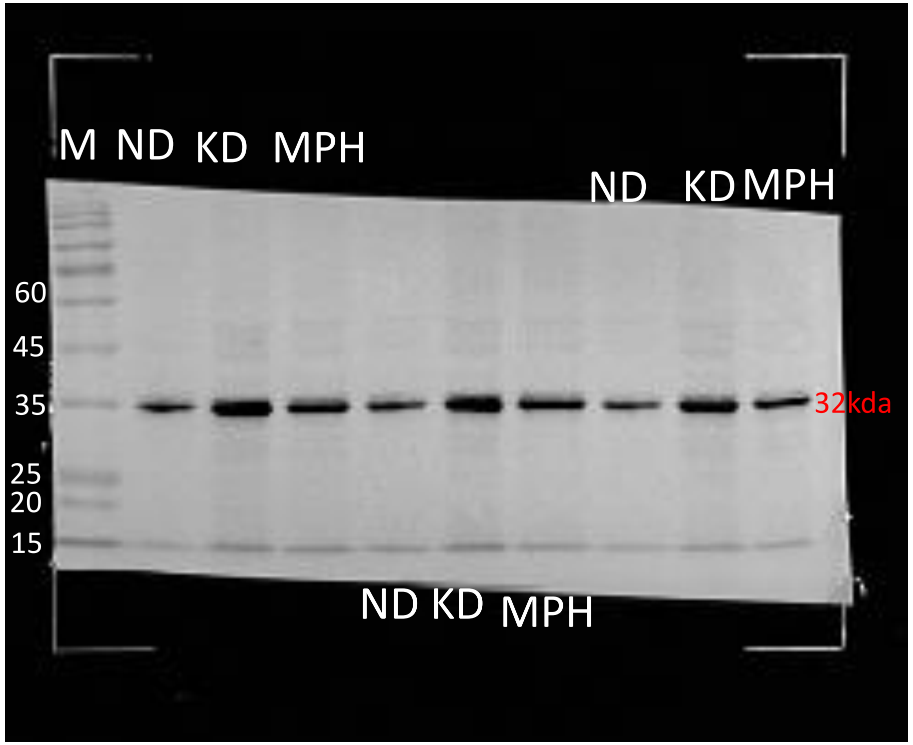

WKY DARPP32

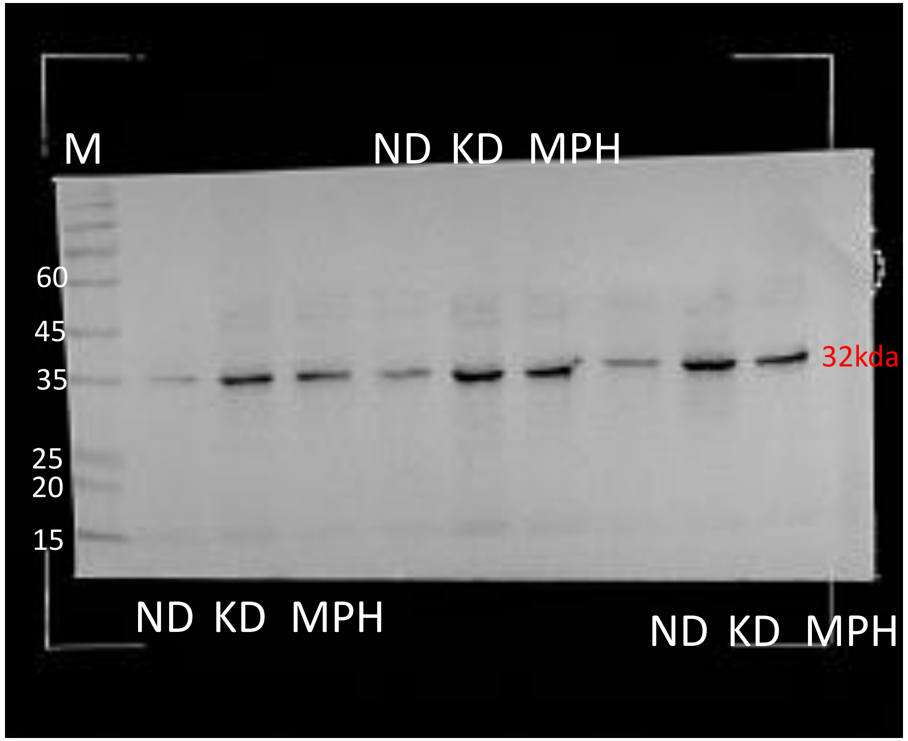

SHR DAT

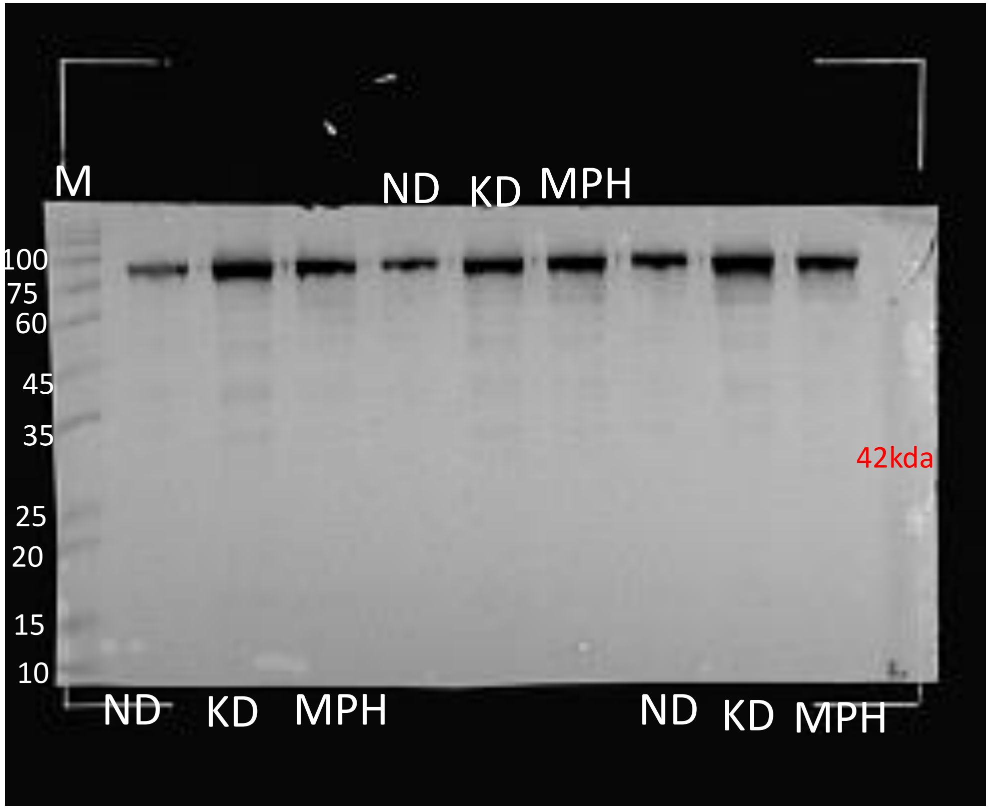

WKY DAT

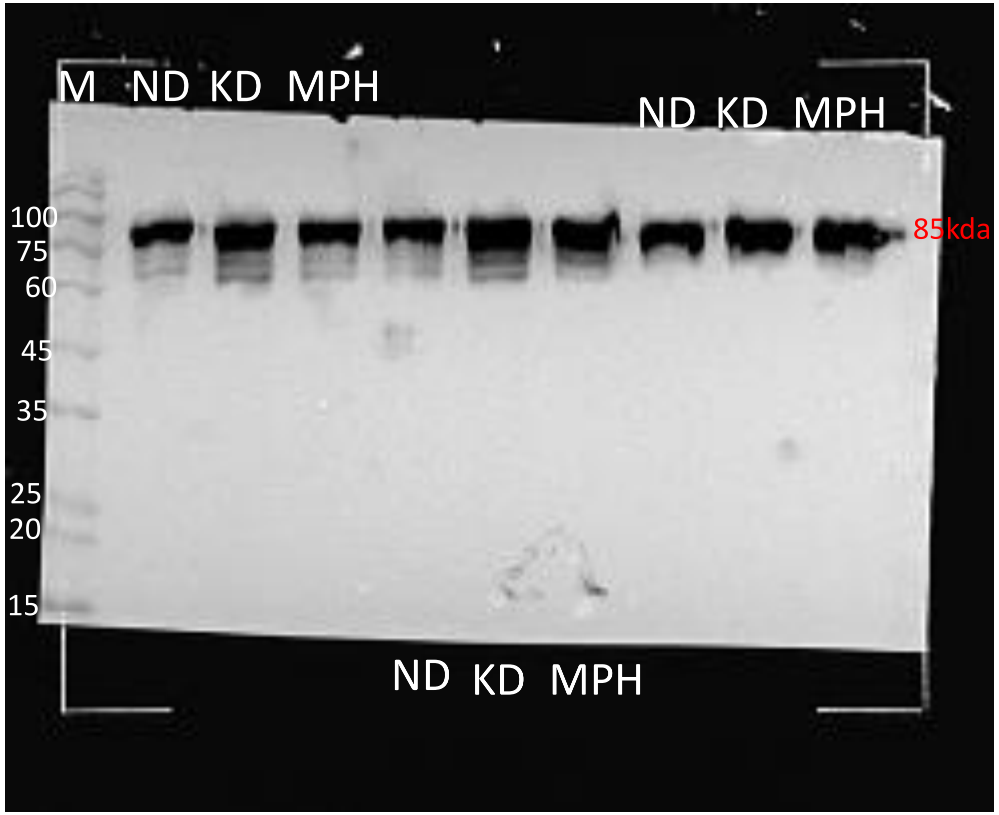

SHR DRD1

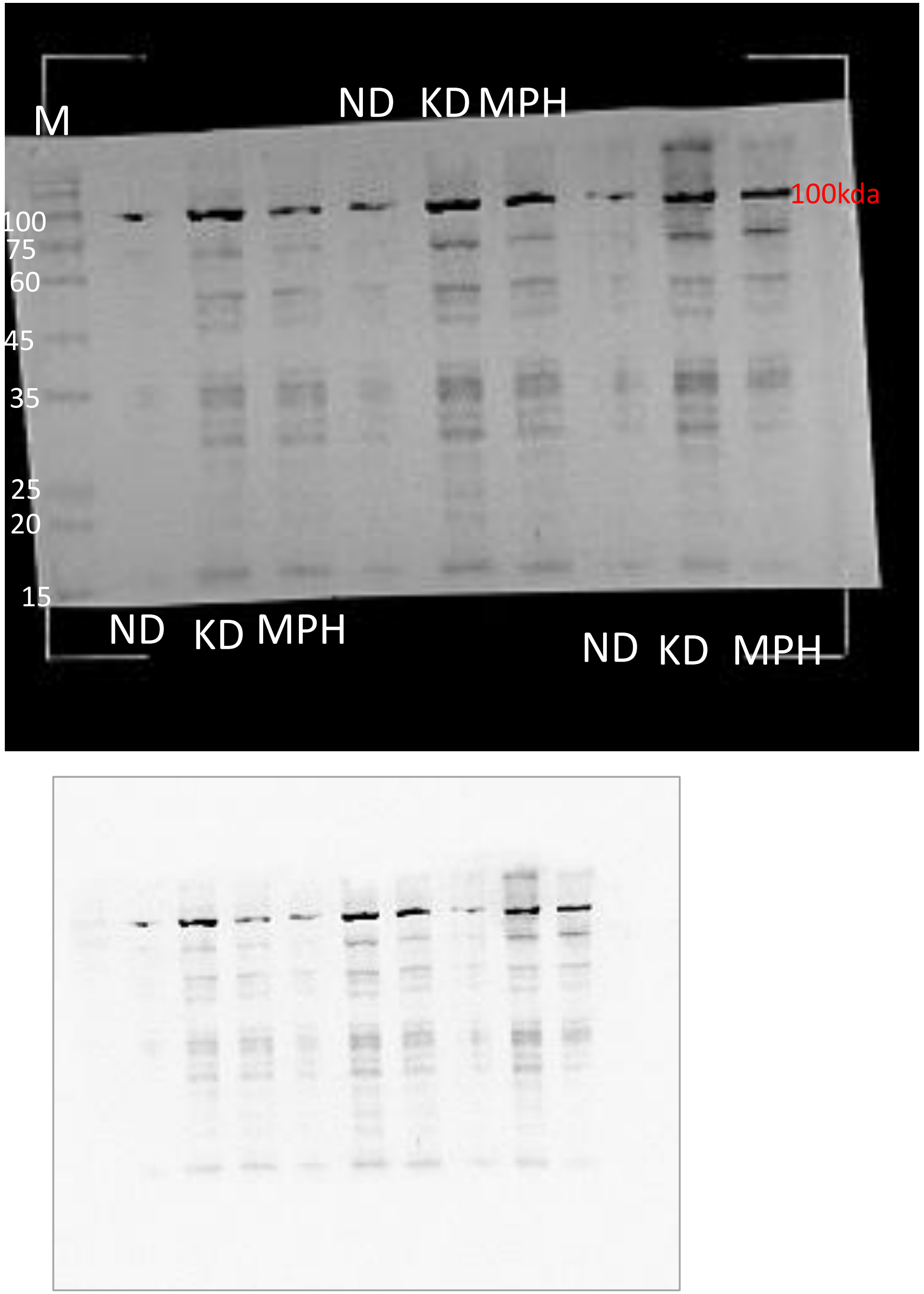

WKY DRD1

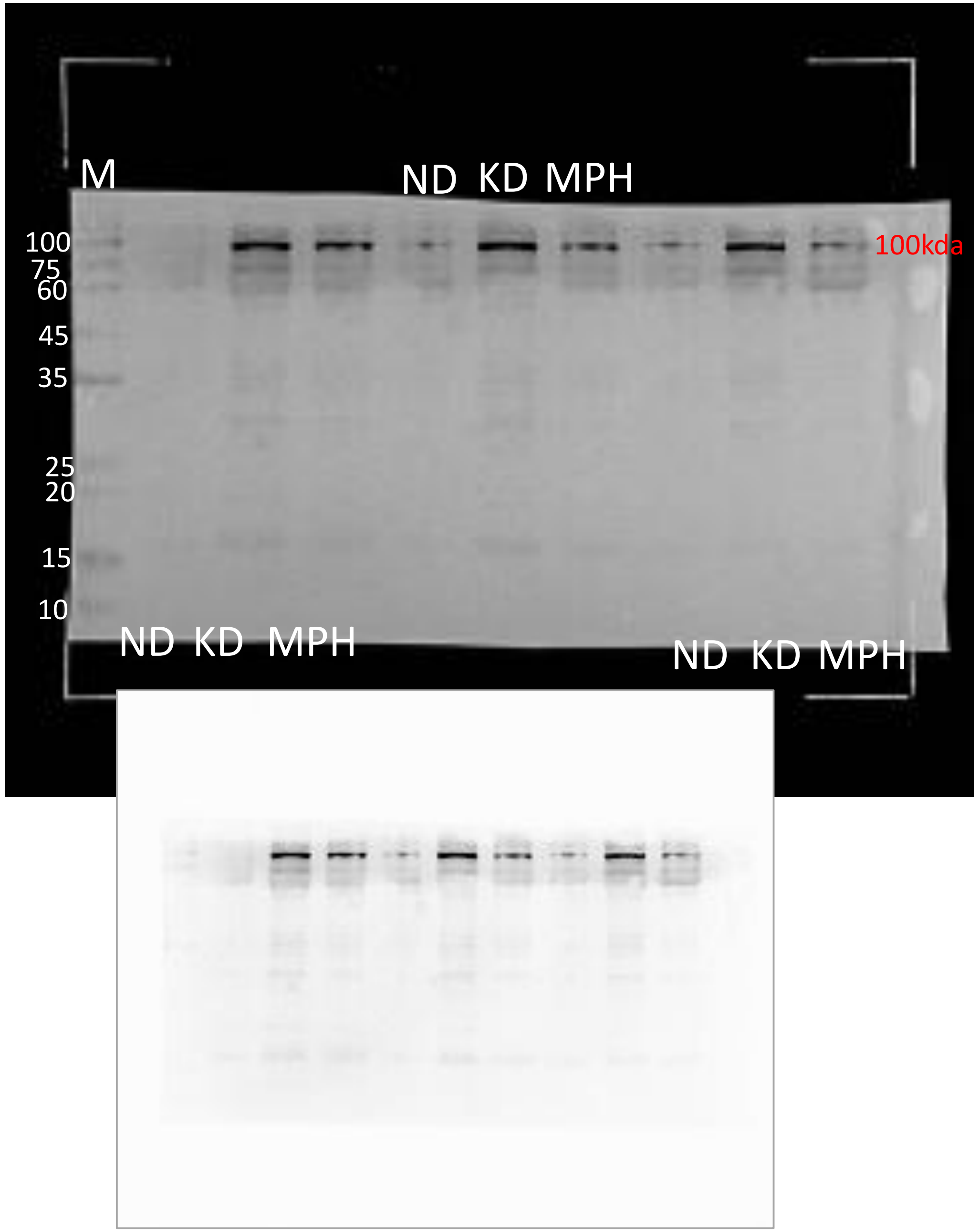

SHR PKA

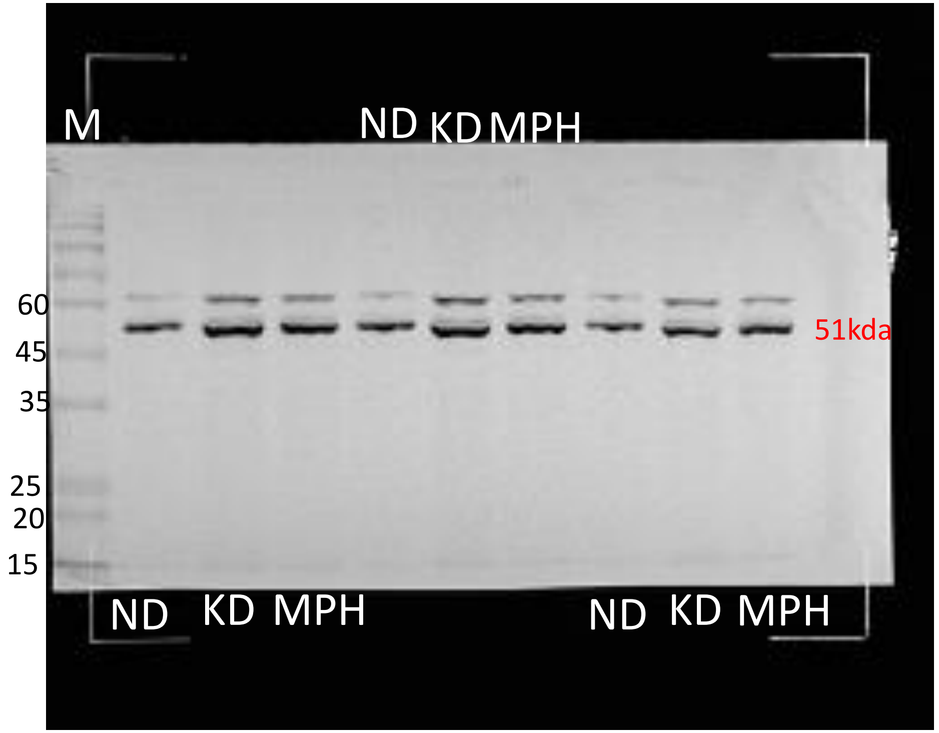

WKY PKA

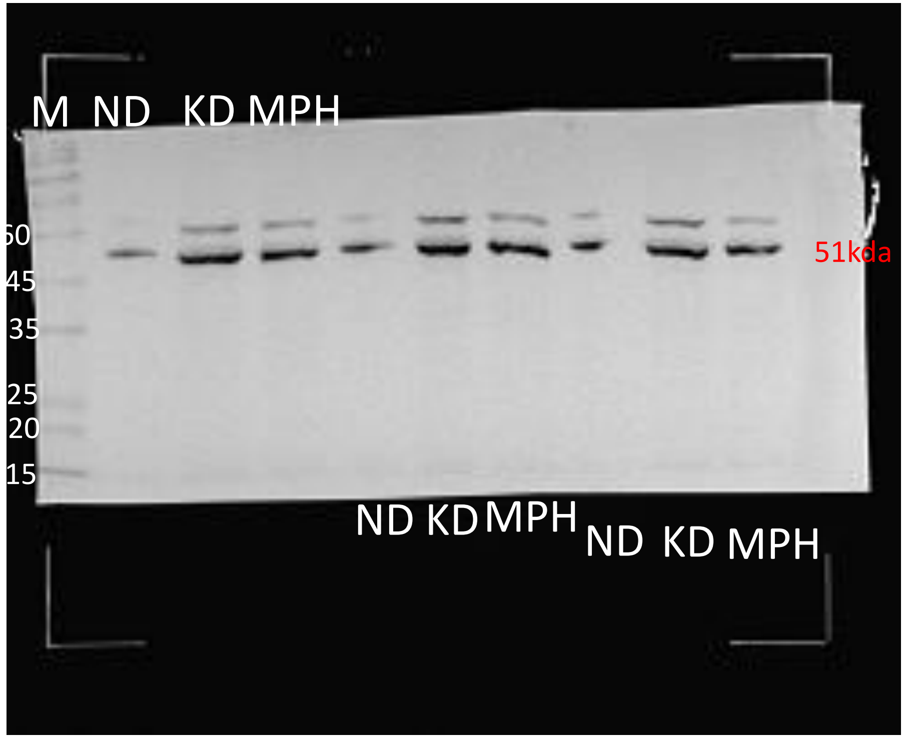

SHR GAPDH

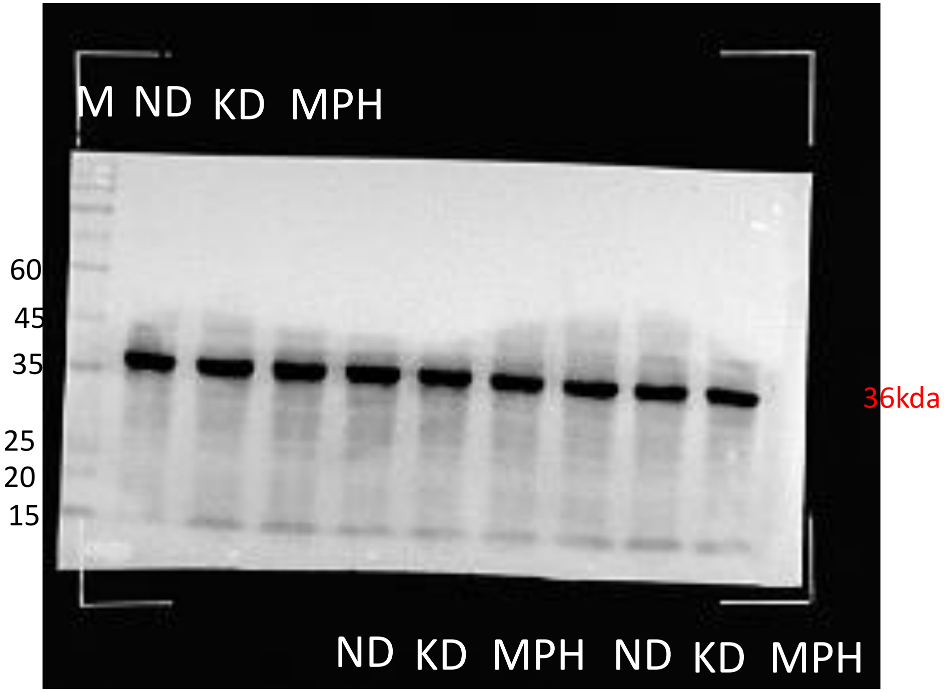

WKY GAPDH

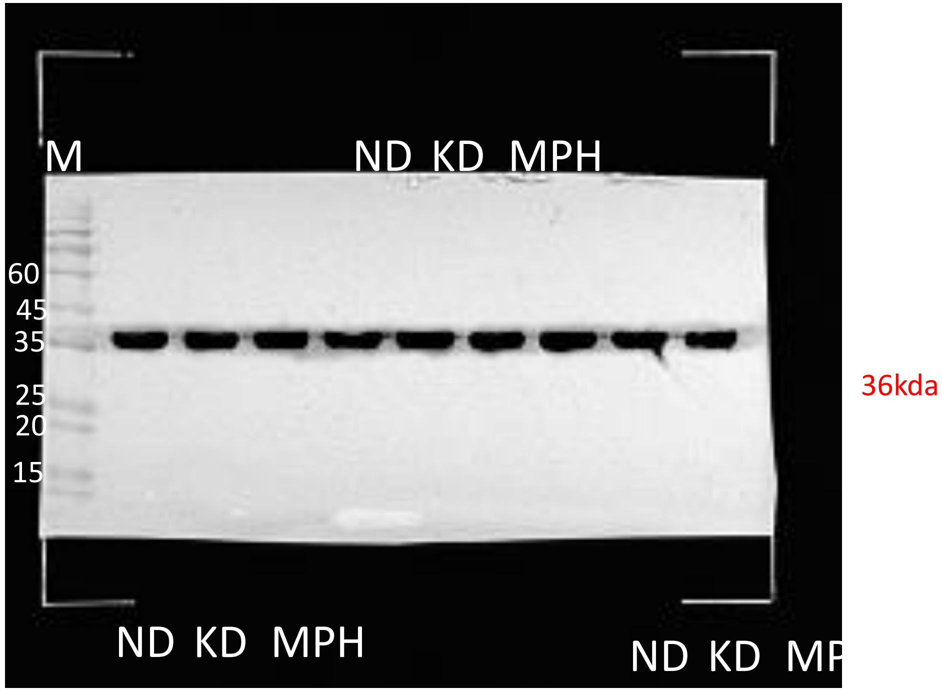

Supplement: S1 Raw image — (PDF) [file pone.0289133.s004.pdf]
